# Fluorescently Labeled Cellulose Nanofibers for Environmental Health and Safety Studies

**DOI:** 10.3390/nano11041015

**Published:** 2021-04-15

**Authors:** Ilabahen Patel, Jeremiah Woodcock, Ryan Beams, Stephan J. Stranick, Ryan Nieuwendaal, Jeffrey W. Gilman, Marina R. Mulenos, Christie M. Sayes, Maryam Salari, Glen DeLoid, Philip Demokritou, Bryan Harper, Stacey Harper, Kimberly J. Ong, Jo Anne Shatkin, Douglas M. Fox

**Affiliations:** 1 Department of Chemistry, American University, Washington, DC 20016, USA; ilapatel2612@gmail.com; 2 Materials Science and Engineering Division, Materials Measurement Laboratory, National Institute of Standards and Technology, Gaithersburg, MD 20899, USA; jeremiah.woodcock@nist.gov (J.W.); ryan.beams@nist.gov (R.B.); stephan.stranick@nist.gov (S.J.S.); ryan.nieuwendaal@nist.gov (R.N.); jeffrey.gilman@nist.gov (J.W.G.); 3 Department of Environmental Science, Baylor University, Waco, TX 76798, USA; Marina_George@baylor.edu (M.R.M.); Christie_Sayes@baylor.edu (C.M.S.); 4 Department of Environmental Health, Center for Nanotechnology and Nanotoxicology, T.H. Chan School of Public Health, Harvard University, Boston, MA 02115, USA; msalari@hsph.harvard.edu (M.S.); gdeloid@hsph.harvard.edu (G.D.); pdemokri@hsph.harvard.edu (P.D.); 5 Department of Environmental and Molecular Toxicology, Oregon State University, Corvallis, OR 97331, USA; bryan.harper@oregonstate.edu (B.H.); stacey.harper@oregonstate.edu (S.H.); 6 Vireo Advisors, LLC, Boston, MA 02130, USA; kong@vireoadvisors.com (K.J.O.); jashatkin@vireoadvisors.com (J.A.S.)

**Keywords:** cellulose nanomaterials, fluorescence, trace labeling, toxicity

## Abstract

An optimal methodology for locating and tracking cellulose nanofibers (CNFs) in vitro and in vivo is crucial to evaluate the environmental health and safety properties of these nanomaterials. Here, we report the use of a new boron-dipyrromethene (BODIPY) reactive fluorescent probe, meso-DichlorotriazineEthyl BODIPY (mDTEB), tailor-made for labeling CNFs used in simulated or in vivo ingestion exposure studies. Time-correlated single photon counting (TCSPC) fluorescence lifetime imaging microscopy (FLIM) was used to confirm covalent attachment and purity of mDTEB-labeled CNFs. The photoluminescence properties of mDTEB-labeled CNFs, characterized using fluorescence spectroscopy, include excellent stability over a wide pH range (pH2 to pH10) and high quantum yield, which provides detection at low (μM) concentrations. FLIM analysis also showed that lignin-like impurities present on the CNF reduce the fluorescence of the mDTEB-labeled CNF, via quenching. Therefore, the chemical composition and the methods of CNF production affect subsequent studies. An in vitro triculture, small intestinal, epithelial model was used to assess the toxicity of ingested mDTEB-labeled CNFs. Zebrafish (*Danio rerio*) were used to assess in vivo environmental toxicity studies. No cytotoxicity was observed for CNFs, or mDTEB-labeled CNFs, either in the triculture cells or in the zebrafish embryos.

## 1. Introduction

Cellulose is the most abundant renewable, biodegradable and non-toxic biopolymer on earth. The general term “cellulose nanomaterials (CNMs)” refers to cellulosic extracts or processed materials, having defined nano-scale structural dimensions [1,2]. CNMs are readily available in the market as a commercial product and their areas of application span across the paper, paint, construction, biocomposites, electronics, tissue engineering, drug delivery, food and packaging industries [1,3]. These nanoparticles exhibit exceptional features such as nano morphology in at least two dimensions, high aspect ratios, large surface areas, low densities, tunable surface functionalities and unique rheologies [4,5]. Recently, CNMs have garnered increasing attention due to their biodegradability, biocompatibility and abundance of hydroxyl groups, which are easily modified by the grafting of different molecules [6]. However, owing to the nanoscale features, the environmental health and safety (EHS) of CNMs is an important issue that needs to be assessed.

Typically, CNMs are classified into two major groups: cellulose nanocrystals (CNCs), and cellulose nanofibers (CNFs). Variations in CNMs generally arise from three factors: (i) the cellulose source (plants, tunicates, bacteria) (ii) extraction/production method (pretreatments step) and (iii) surface chemistry (functionalization). CNMs extracted from plants are prepared by a variety of pretreatment steps followed by various refinement steps [7]. Refinement by acid hydrolysis of the cellulose fibers preferentially cleaves the chains at the disordered regions of the cellulose and gives rod-shaped crystals, CNCs, with dimensions of 2 nm to 20 nm diameters and 100 nm to 500 nm lengths. The CNCs contain highly crystalline cellulose domains, ranging from 54% to 88% crystallinity [4]. In contrast, refinement by mechanical treatments uses high shear forces to comminute the cellulose source material into CNMs, and the resulting particles are designated as either microfibrillated cellulose (MFC) or CNFs, depending on the fibrillation extent. MFC contains significant amounts of micron-sized particles (in at least two dimensions) and are not a part of this study. CNFs exhibit the highest extent of fibrillation and are micrometer long fibrils or fibers with diameters on the nanometer scale (typically <50 nm) that contain both amorphous and crystalline cellulose domains. These fibers exhibit large specific surface areas, extensive hydrogen bonding between fibers, and a high degree of entanglement. This gives rise to highly viscous aqueous suspensions at relatively low concentrations (below 1% by mass) [4,7]. CNFs also contain trace amounts of lignin, hemicellulose, or fragments of these contaminants.

Until recently, the environmental and health impacts of CNFs have remained largely unexplored. Increased interest in commercialization has prompted progress towards characterizing the EHS of CNFs, both in the workplace and in potential products. The potential use of CNFs in medical, food, and packaging applications also requires examination of EHS by ingestion. Some of the potential implications of CNF ingestion exposures have recently been studied and reported in the literature. These studies have included investigations of the toxicity of physiologically relevant pre-digested CNF in an in vitro model of the small intestinal epithelium [8,9], as well as in vivo systemic toxicity, intestinal epithelial health effects, and impacts on the gut microbiota in rats [8,10,11]. Other than a reported significant reduction in digestion and absorption of co-ingested triglycerides (fat) [12], which may provide a health benefit, toxicology results to date generally have not reported any significant adverse effects. One critical study, however, remains elusive: the measurement and reporting of the absorption, distribution, metabolism, and excretion (ADME), or, i.e., the toxicokinetic characteristics, of CNFs, which is critical for demonstrating the safety of oral exposure. Such studies require the ability to accurately quantify CNFs in biological media in order to determine the ADME by combined direct measurements and mass balance calculations.

The recommended guideline protocol for studying the safety of carbohydrates is radioactive carbon (^14^C) labeling and tracing [13,14,15]. The ^14^C traces are then detected and quantified in various organs of an exposed rodent test model to establish toxicokinetic profiles, which are then used in physiologically based toxicokinetic models to assess safety. However, the use of radioactive carbon is dangerous to occupational workers and the amount of ^14^C within a test substance is miniscule. This requires highly specialized instrumental resources (personnel and facilities), which are often cost-prohibitive. Fluorescent labeling is a viable alternative safety testing strategy that should be further advanced and used in iterative product development. It has already been successfully used to track polymer uptake into cells and should also provide an alternative to other in vivo studies [16,17].

Fluorescence offers several advantages as a label for these types of studies: it uses a fairly simple labeling process, there are a range of fluorophores available to avoid interference, it can be used to examine interfaces, and imaging is available in most laboratories. There are some disadvantages that must be considered: pH and photo stability, detection limits, chemical stability, and interferences with biological autofluorescence. These disadvantages can be avoided by choosing the appropriate fluorophore. The requirements for an ADME test are that the material must be representative of commercial cellulose nanomaterial, the label must remain attached to CNM during digestive process, the CNM needs to be detectable at various stages of digestion, it must be detectable at high dilution (1–10 μg/mL), and the fluorophore must not have any biological fluorescence interferences. The range of appropriate fluorophores and attachment chemistries used to produce fluorescently labeled CNFs for ADME studies is significantly narrowed by these conditions. The reaction must be performed in water to avoid aggregation. The reaction steps and extent of labeling must be minimized to prevent changes in surface chemistry or surface energy. The labeling bond must not dissociate between pH 1 and pH 8 or in the presence of digestive enzymes. Additionally, the fluorophore must have an emission with high quantum yield, invariable quantum yield over pH range, and an excitation λ > 500 nm. A wide variety of fluorogenic dyes have been used to label CNMs [18,19,20,21]. However, none of the strategies used to date meet all of these criteria.

**Figure 1:**
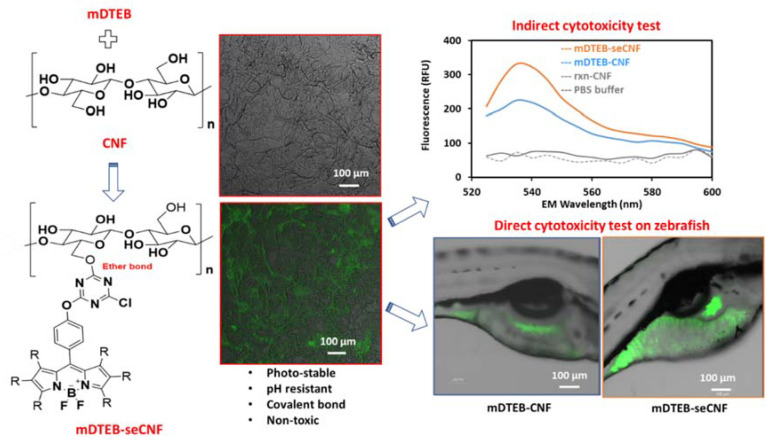
Study overview: cellulose nanofiber (CNF) was labeled with meso-DichloroTriazineEthyl boron-dipyrromethene (BODIPY) (mDTEB). The labeled material was characterized for changes in surface properties and morphology. The labeled materials were subsequently tested for in vivo and in vitro toxicity.

The aim of the current study was to synthesize chemically and enzymatically stable, fluorescently labeled CNFs that meet all the criteria needed for ADME studies and to study specific cytotoxicity effects and biodistribution in vivo and in vitro after ingestion (Figure 1). The notation used for the various materials utilized in this study are summarized in Table 1.

## 2. Materials and Methods

Research carried out at the National Institute of Standards and Technology (NIST), an agency of the U.S. government and by statute is not subject to copyright in the United States. Certain commercial equipment, instruments, materials or companies are identified in this paper in order to adequately specify the experimental procedure. Such identification is not intended to imply recommendation or endorsement by the National Institute of Standards and Technology, nor is it intended to imply that the materials or equipment identified are necessarily the best available for this purpose.

*Materials.* Mechanically produced cellulose nanofibers (3.0% by mass, 90% by mass fines, never-dried) were provided by the University of Maine Process Development Center, Orono, ME nanocellulose pilot plant. The boron-dipyrromethene (BODIPY) fluorescent probe, meso-DichloroTriazineEthyl BODIPY (mDTEB), was synthesized according to the literature procedure [22]. Deionized water (18.2 MΩ, Millipore Milli-Q Purification System) was used throughout. All solvents used in the test were chromatographically pure. Na_2_CO_3_, NaOH, and buffer salts were obtained from Sigma–Aldrich. The pH value of the buffer was measured with a Fisher Science Education Benchtop pH meter (Fisher Scientific).

*Physico-chemical characterization of CNF.* The chemical composition of the CNF was analyzed according to NREL (National Renewable Energy Laboratory) procedures [23], and determined to consist of 96.0% by mass cellulose, 1.5% by mass hemicellulose and 2.5% by mass lignin. The total lignin content was determined from CNF samples according to TAPPI (Technical Association of the Pulp and Paper Industry) T222 om-06 [24] and TAPPI UM250 [25], to analyze the acid-insoluble residues (AIRs) and acid-soluble lignin (ASL), respectively. Briefly, CNF was hydrolyzed using sulfuric acid (72% by mass) for 1 h at 30 °C. After hydrolysis, samples were diluted (3% by mass) in deionized water and autoclaved at 121 °C for 1 h. The resulting solutions were cooled to room temperature and the precipitates were filtered and dried. The mass of the precipitate was designated as the AIR content (also known as “Klason lignin”). The ASL content was calculated by measuring absorbance at 205 nm with a spectrophotometer. The total yield of lignin is presented in Figure S1. Surface impurities of CNF was examined by a confocal laser scanning microscopy (CLSM) with a 20×, 0.7 numerical aperture (NA) air objective. Additional information about the photoluminescence properties of CNF-extracted impurities was obtained using fluorescence spectroscopy.

*Preparation of surface extracted CNF.* As received CNF (3.0% by mass) was diluted by adding 0.1 M NaOH to obtain 1.5% by mass CNF. The diluted CNF suspension was autoclaved at 105 °C for 15 min and centrifuged at 520 rad/s (12,500× *g*) for 10 min to separate solid and liquid fractions. Analysis of the liquid fraction was performed using a fluorimeter. The solid fraction was washed with water until a neutral pH was reached. Cleaned, extracted CNF with a final concentration between 2.5% and 4% by mass was designated as surface extracted CNF (seCNF) and stored at 4 °C until needed for further analysis or experiments.

*Synthesis of mDTEB-labeled CNF.* Either as received CNF slurry or seCNF slurry. (enough to contain 4.5 g dry mass) and 100 mL of water was added to 150 mL of 50 mM Na_2_CO_3_ and mechanically stirred for 30 min. 2 mg of mDTEB was dissolved in 500 µL of acetone, then added to the alkaline CNF suspension and stirred for 72 h in the dark at room temperature. When the reaction was complete, the modified CNF was isolated by centrifugation at 520 rad/s (12,500× *g*) for 20 min. After centrifugation, the excess of mDTEB was removed by washing the labeled CNF with an ethyl acetate—water mixture (washing solvent). Purification was carried out using a Speed Mixer (DAC 400 Mixer range, FlackTek INC, Landrum, SC, USA) at 150 rad/s for 10 min followed by centrifugation at 420 rad/s (10,000× *g*) for 10 min. The purification step was repeated until no fluorescent signal was detected in the washing liquor. The labeled CNF was resuspended in water and centrifuged repeatedly until the ethyl acetate was completely removed. Cleaned, labeled CNF was stored at 4 °C until needed for the further analysis. Based on fluorescence differences of filtered reaction fluids (0.2 mm PTFE syringe disc) before and after the reaction, it is estimated that the degree of labeling was 0.3 μmol/g CNF and 0.2 μmol/g seCNF, which is equivalent to about 1 every 20,000 to 30,000 anhydrous glucose units.

*Assessment of photoluminescence properties of labeled CNF.* Photoluminescence excitation (PLE) spectroscopy maps and line scans were obtained using a JY-Horiba Nanolog-3 spectrofluorometer and were corrected for the instrument’s source spectral distribution and detector spectral response. Excitation wavelengths were scanned in 5 nm increments using a 450 W xenon lamp through a 2 nm slit. Emitted light was collected at 90° and measured using a liquid N_2_-cooled InGaAs detector over 2 nm increments through a 2 nm slit. Solution samples were placed in a 2 mm pathlength quartz cuvette and solid samples were analyzed by spin coating and drying solutions onto glass cover slips. The fluorescence emission spectra of mDTEB-CNF and mDTEB-seCNF were created using an excitation wavelength (λ_Ex_) of 514 nm with emission wavelengths from 530 nm to 650 nm. An excitation plot was created with an emission wavelength (λ_Em_) of 540 nm with excitation wavelengths from 400 nm to 530 nm. The UV−Vis absorption spectra were collected with a Cary 5000 UV–Vis-NIR spectrophotometer (Full Spectrum Analytics, Inc., Pleasanton, CA, USA). The pH stability of mDTEB-CNF and mDTEB-seCNF were evaluated by measuring fluorescence intensity using a Nanolog-3 spectrofluorometer. Buffered solutions containing 0.5 mg/mL labeled CNF in the range of pH 2 to 10 were equilibrated to 37 °C, and the fluorescence intensity was measured at λ_Em_ 540 nm. The thermal stability of mDTEB-CNF and mDTEB-seCNF were studied by autoclaving 0.5 mg/mL labeled CNF in an alkaline solution (0.1 M NaOH) at 105 °C for 15 min. Unlabeled materials suspended at the same concentration in the same solutions were used as controls. A sample of the fibers were removed, washed with de-ionized water, and spin coated onto glass cover slips. The supernatant was filtered through a 0.2 mm syringe filter and added to a cuvette. A schematic is shown in Figure S2. The photoluminescence spectra of autoclaved washed fibers, the collected supernatants, and the controls were recorded using the spectrofluorometer as described above. The concentration-dependence of the labeled CNF fluorescence was assessed by measuring the fluorescence intensity of labeled CNF diluted to concentrations between 2 µg/mL and 2 mg/mL using PBS (phosphate – buffered saline) buffer or deionized water. Fluorescence intensity was recorded on a Tecan Spark M20 multimode microplate reader equipped with monochromator optics (Tecan Group Ltd., Männedorf, Switzerland) at λ_Ex_ 514 nm and λ_Em_ 540 nm. Measurements were performed with top well illumination, using black/clear bottom 96-well micro-plates. Each suspension was measured in triplicate using 200 μL samples. Intensity values were background corrected. For fluorescence images, dilute labeled CNF suspensions were deposited onto an ultraviolet ozone (UVO)-treated glass slides and dried at room temperature. Confocal microscopy measurements were carried out using an upright Leica TCS SP5 II (Leica Microsystems, Buffalo Grove, IL, USA), with a 20×, 0.7 numerical aperture (NA) air objective. Images were acquired by exciting at 488 nm and collecting emission at 500 nm to 650 nm.

*Microscopy images of CNF.* The fluorescence lifetime imaging microscopy (FLIM) of CNF samples were determined using a time-resolved single photon counting module (SPCM) set up [26]. CNF samples were drop-cast onto ultraviolet ozone (UVO)-treated glass slides, dried at room temperature, and imaged using two-photon fluorescence. The images presented are 256 × 256 pixels with dwell times of a 2 ms. Wide-field optical microscopy images were collected using a DMi 1 Leica Microsystems Inverted Microscope. Samples were diluted to 0.1% by mass and 1 drop was placed on a glass microscope slide. The sample was spread and allowed to air dry before imaging in bright-field mode. Scanning electron microscopy (SEM) images of CNF were obtained on a Focused Ion Beam Scanning Electron Microscope Versa 3D, FEI Company with an acceleration voltage of 5.00 kV using either an ETD (Everhart—Thornley Detector). Samples were prepared by first drying an aliquot of suspended cellulose on an SEM puck and pin stub. The sample was then sputter coated with a 5 nm layer of carbon to decrease sample charging.

*Inverse gas chromatography (iGC).* Surface energy analyses were carried out using an iGC surface energy analyzer (SEA) (Surface Measurement Systems, Alperton, UK) and the data were analyzed using both standard and advanced SEA Analysis Software. iGC surface energy measurements and analysis calculations were conducted at finite dilutions according to the published procedure [27,28,29]. Briefly, approximately 150 mg of freeze-dried CNF was packed into individual silanized glass columns. The BET surface area was determined using a linear regression of n-octane surface coverage between 0.05 and 1.0 fractional coverage. Each column was conditioned and dried under a flow of anhydrous helium for 30 min, before n-alkane probe molecules (hexane, nonane, octane, and heptane) and polar probe molecules (acetone, ethanol, acetonitrile, ethyl acetate, and dichloromethane) were introduced over a range of injection volumes. This method was used to determine the dispersive and acid−base components of surface energy as a function of fractional surface coverage. This method also permits the investigation of energetic heterogeneity. The estimated error in BET surface area and surface energies at 0.05 fractional coverage are estimated to be 2σ = ±1.3 m^2^/g and 2σ = ±0.3 mJ/m^2^, respectively.

*Dynamic Light Scattering/Zeta Potential (DLS/ZP).* A Malvern Instruments Zetasizer Nano ZS running on Zetasizer Software 7.10 was used to characterize the average particle sizes and surface charges of the CNM. Samples were placed in 20 mL scintillation vials and diluted to 0.1% by mass in ultrapure water. Dilutions of CNF were prepared in triplicate and measured in triplicate (9 total measurements for each material tested) using 1 mL sample suspensions dispensed in disposable microcuvettes (Cuvette Pack with Stoppers (ZEN0118), Malvern Inc., Westborough, MA, USA). An absorbance of 0.01 and refractive index of 1.580 were used for CNF and a viscosity of 0.8872 cP and refractive index of 1.330 were used for the dispersant (water). DLS reports an intensity-weighted averaged hydrodynamic diameter (D50) and polydispersity index (PDI), which is a dimensionless measure of particle size heterogeneity. As DLS can only provide accurate size measurements for nearly spherical values, for suspensions of anisotropic materials with high aspect ratios, such as CNF, D50 values indicate only relative particle sizes rather than true hydrodynamic diameters.

*Endotoxin and sterility assessment of CNF materials.* All CNF materials were tested for endotoxin using the EndoZyme^®^ recombinant factor C (rFC) assay (Hyglos, Germany) according to the product manual’s instructions, as previously described [30]. Briefly, 10 µg/mL suspensions of CNFs, as well as endotoxin standard dilutions, and ENM suspensions spiked with 0.5 EU/mL endotoxin, were prepared in endotoxin-free water. Samples, spiked samples, and standard dilutions were dispensed into a pre-warmed (37 °C) 96 well plate (100 µL/well) and mixed with 100 µL of assay reagent (8:1:1 ratio of assay buffer, enzyme, and substrate). Fluorescence (λ_Ex_ = 380 nm, λ_Em_ = 440 nm) was measured at t = 0 min and 90 min. Endotoxin levels were calculated from sample fluorescence using a standard curve equation generated from a range of endotoxin standard dilutions.

Microbiological sterilities of all CNF materials were assessed using the WHO protocol in the international pharmacopoeia as previously described [30]. Briefly, CNF materials were suspended at 1 mg/mL, and 1 mL of each suspension was added to 10 mL of liquid thioglycolate medium at pH 6.9 to 7.3. The resulting solutions were incubated at 37 °C for 14 d and examined daily for indications of bacterial growth. Every three days during incubation, samples of broth were spread onto tryptic soy agar plates and mixed with either potato dextrose agar or plate count agar to create pour plates. These plates were incubated at 37 °C for 3 days and then examined for growth of bacterial and fungal colonies.

*In vitro simulated digestion.* In vitro simulated digestion was performed using a 3-phase simulator as previously described by the authors [31]. CNFs were added to a fasting food model (5 mM phosphate buffer) at 0.75% or 1.5% by mass. CNFs have not proceeded through regulatory review as food additives, and no guidelines for concentrations exist. However, microcellulose materials are considered generally recognized as safe (GRAS) food additives in the U.S. There are no limits placed on the amounts in food except in some meat and poultry products, where powdered cellulose is allowed up to 3.5% by mass, and microcrystalline cellulose up to 3.0% by mass. However, given the likely greater surface areas of nanocellulose materials, the high viscosity of CNF suspensions at greater than 1.0% by mass, and the reported efficacy of CNF in food applications at 0.2% to 1.0% by mass [32] realistic usages would be unlikely to exceed 1% by mass. Concentrations of 1.5% and 0.75% by mass were thus chosen to bracket this value. Food–nanomaterial mixtures were combined with equal volumes of simulated saliva fluid, and incubated for 2 min. The resultant mouth digesta was then combined with simulated gastric fluid and incubated for 2 h at 37 °C in an orbital shaker. The resulting gastric phase digesta was combined with additional salts, bile extract and lipase to simulate intestinal fluid, and incubated at 37 °C for 2 h while maintaining a constant pH of 7.0 using a TitroLine 7000 pH Stat titration device (SI Analytics, GmbH, Mainz, Germany).

*In vitro toxicity assessment in a tri-culture model of the small intestinal epithelium.* The in vitro tri-culture small intestinal epithelial model has previously been characterized and described in detail [31]. All cell lines were obtained from Sigma–Aldrich Corp (St. Louis, MO, USA). Caco-2 and HT29-MTX cells were grown in high-glucose Dulbecco’s Modified Eagle Medium (DMEM) supplemented with 10% by mass heat-inactivated fetal bovine serum (FBS, Sigma–Aldrich), 10 mM HEPES (N-2-hydroxyethylpiperazine-N-2-ethane sulfonic acid) buffer, 100 IU/mL penicillin, 100 µg/mL streptomycin, and non-essential amino acids (1/100 dilution of 100× solution, Thermo Fisher Scientific, Waltham, MA, USA). Raji B cells were cultured in RPMI 1640 media supplemented with 10% by mass FBS, 10 mM HEPES buffer, 100 IU/mL penicillin and 100 µg/mL streptomycin. To prepare transwell inserts, Caco-2 and HT-29MTX cells were harvested and resuspended in DMEM at 3 × 10^5^ cells/cm^3^ and combined at 3:1 (Caco-2:HT29-MTX). An amount of 1.5 mL of the mixture was dispensed in the apical chamber, and 2.5 mL of complete DMEM media was added to the basolateral compartment of each transwell (Corning, New York, NY, USA). Media was replaced after 4 d, and subsequently every other day, until day 15. On days 15 and 16, basolateral media was replaced with 2.5 mL of 1:1 DMEM: RPMI complete media containing a Raji B cells at 1 × 10^6^ cells/mL. For 96-well plates, 100 µL of the 3:1 mixture of Caco-2 and HT-29MTX cells were dispensed into each well of black-walled, clear bottom plates (BD Biosciences, Billerica, MA, USA), and media was changed after 4 d, and subsequently every other day, until day 17. Toxicology experiments were performed on day 17.

The final small intestinal phase digestase of mDTEB-seCNF and CNF were combined with DMEM in a ratio of 1:3, and the 1.5 mL (transwells) or 200 µL (96-well plates) was applied to test cells. Media in control wells was replaced with fresh complete DMEM media. Cells were incubated for 24 h. Supernatants from transwells were collected for lactate dehydrogenase (LDH) (as a measure of cytotoxicity) analysis, and 96-well plates were processed for viability assessment. LDH was measured using the Pierce LDH assay kit (Sigma–Aldrich, St. Louis, MO, USA) according to manufacturer’s instructions, with untreated control wells used to measure background LDH release, and lysed cells providing maximum LDH release measurements. Percent cytotoxicity was calculated from test, background and maximum LDH controls as recommended by the manufacturer and previously described in detail [8]. Assessment of metabolic activity (cell viability) was performed using the PrestoBlue viability reagent (Thermo Fisher Scientific, Waltham, MA, USA) according to manufacturer’s instructions. Briefly, wells were washed 3 times with 200 µL of PBS, 100 µL of 10% by mass PrestoBlue reagent was added to each well, plates were incubated at 37 °C for 15 min, and fluorescence was measured at λ_Ex_ = 560 nm and λ_Em_ = 590 nm.

*Assessment of* in vivo *Zebrafish toxicity*. To determine acute in vivo toxicity and biodistribution behavior of CNFs, we used an embryonic zebrafish model, as their genetic, cellular, and organ structure is similar to humans [33]. Adult zebrafish (*Danio rerio*) were maintained at the Sinnhuber Aquatic Research Laboratory at Oregon State University. Embryos were collected from group spawns of wild-type 5D zebrafish and staged to ensure all embryos were at the same developmental stage at the start of the experiment. Embryos were enzymatically dechorionated at 6 h post fertilization (hpf) with pronase (Sigma–Aldrich) following the protocol described by Usenko et al. [34]. At 8 hpf, embryos were incubated in batches (n = 50) in 25 mL of either 1 mg/mL mDTEB-CNF, 1 mg/mL mDTEB-seCNF, or fish water (FW). FW was prepared by mixing 0.26 g/L Instant Ocean salts (Aquatic Ecosystems, Apopka, FL, USA) in reverse osmosis water and adjusting the pH to 7.2 ± 0.2 with sodium bicarbonate. Conductivity was between 480 μS/cm to 520 µS/cm. At 120 hpf the zebrafish were rinsed three times with FW, then imaged by confocal microscopy (λ_Ex_ 504 nm, λ_Em_ 528 nm). Protoslo (Carolina Biological Supply Company, Burlington, NC, USA) was added to each deep well to ensure that the fish remained still while being imaged.

## 3. Results and Discussion

### 3.1. Fluorescently Labeled Cellulose

The development of a reactive fluorescent label for cellulose nanomaterials for ingestion exposure studies requires careful consideration. The labeled CNM must be representative of commercial cellulose nanomaterials, remain attached to the cellulose during the digestive process, be detectable at various stages of digestion, be detectable at high dilution (~1 μg/mL), and not subject to interference from biological fluorescence sources. As noted in the introduction, these criteria eliminate nearly every labeling chemistry used previously, including the popular use of isothiocyanates and most commercially available dyes. To meet all of the requirements for digestive studies of CNM, it was therefore necessary to design and synthesize a new fluorophore. A 4,4-difluoro-4-bora-3a,4a-diaza-s-indacene (BODIPY) was chosen as the fluorescent center because this family of dyes are known to have high quantum yields, chemical stability, photostability, and lack of toxicity [35,36,37]. A considerable volume of work involving BODIPY fluorophores has focused on their use as labeling reagents for biological materials [36,37,38,39,40,41]. Alkyl substitutions were added to the fluorescent center to increase the excitation wavelength above 490 nm. The moiety in the meso position of the fluorescent center was functionalized with a chlorotriazine. This functional group can react easily with nucleophiles such as alcohols or amines under mildly basic conditions to yield a stable aromatic ether linkage. Synthesis and characterization of the new fluorescent dye is detailed elsewhere [22].

Using this new fluorophore, a single pot chemical reaction was performed to synthesize fluorescently labeled CNF. The BODIPY-based fluorescent probe (mDTEB) was covalently attached to CNF using an etherification reaction in the presence of sodium carbonate, resulting in an ether bond formation between the triazine moiety of mDTEB and hydroxyl group of CNF. This bond is stable over a wide range of pH and was expected to survive the digestive process.

**Figure 2:**
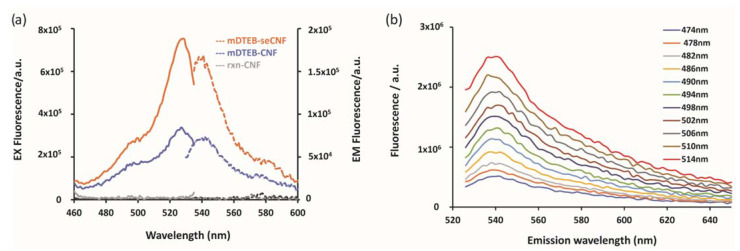
Fluorescent properties of mDTEB dye and mDTEB-seCNF: (**a**) normalized fluorescence spectroscopic data of mDTEB-seCNF compared with the free dye (mDTEB) and (**b**) photoluminescence emission spectra of mDTEB-seCNF. All samples were measured at 0.5 mg/mL CNF.

The photoluminescence properties (excitation and emission spectra) of the mDTEB dye and mDTEB-seCNF were recorded using fluorescence spectroscopy. A maximum excitation intensity was obtained at a wavelength of 530 nm for mDTEB-seCNF (Figure 2a, dotted line), while the maximum emission intensity was obtained at a wavelength of 540 nm (Figure 2a, solid line). Similar excitation λ_max_ and emission λ_max_ were observed for mDTEB-CNF, but with lower fluorescence intensities at the same concentration of CNF (500 µg mL^−1^). The small Stokes shift of 10 nm is one of the few disadvantages to using BODIPY dyes and can limit the choice of fluorescence measuring instruments for some studies. To determine whether or not the synthesized mDTEB-seCNF exhibited excitation-dependent photoluminescence, emission spectra were collected for a range of excitation wavelengths. Figure 2b shows the photoluminescence emission spectra of mDTEB-seCNF with excitation wavelengths from 474 nm to 514 nm. These results illustrate that the mDTEB-CNF produces strong fluorescence emissions with symmetrical emission peaks and an increase in fluorescence emission as the excitation wavelength increases from 474 nm to 514 nm.

**Figure 3:**
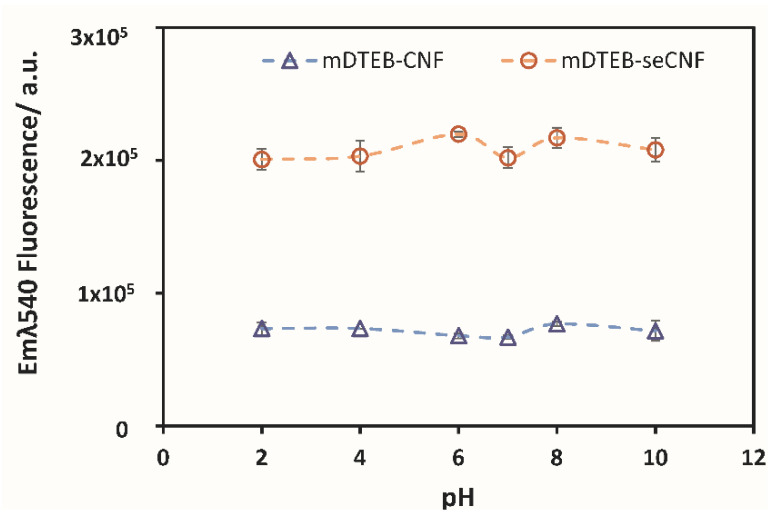
The pH dependence fluorescence of labeled CNF (circle: mDTEB-seCNF, triangle: mDTEB-CNF) at 0.5 mg/mL in buffered solutions.

The effect of pH and temperature on mDTEB-CNF and mDTEB-seCNF were examined fluorometrically. Figure 3 shows stable fluorescence intensity over a wide pH range, from acidic to alkaline conditions, reflecting the stability of mDTEB-labeled CNF at different pH. The stability is observed for both mDTEB-CNF and mDTEB-seCNF. Buffer types (chloride, citrate, phosphate, and glycine) did not affect the fluorescence of the labeled CNF. mDTEB-seCNF exhibited higher intensities compared to mDTEB-CNF at similar concentrations, indicating that surface impurities impact fluorescent labeling of CNF.

**Figure 4:**
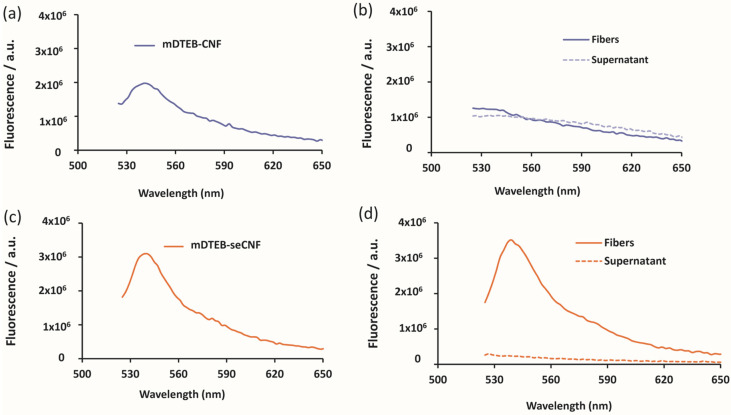
Temperature stability of mDTEB-CNF (**a**) fluorescence of mDTEB-CNF before autoclave, (**b**) fluorescence of mDTEB-CNF after autoclave, (**c**) fluorescence of mDTEB-seCNF before autoclave, (**d**) fluorescence of mDTEB-seCNF after autoclave. Fibers measured as solid samples mounted on cover slips and supernatant measured on filtered samples containing 0.5 mg/mL CNF.

The temperature stability of the ether bond linkages under alkaline condition was assessed by autoclaving the labeled CNF at 105 °C for 15 min. Many biological materials are sterilized prior to use and these conditions are typical for sterilized samples. Figure 4 shows the emission spectra of labeled CNF before and after autoclaving. For mDTEB-CNF, the fluorescence intensity of the fibers after washing was significantly reduced after the alkaline treatment (Figure 4b). There was a corresponding increase in the fluorescence of the supernatant. On the other hand, mDTEB-seCNF exhibited no loss in fluorescence intensity after autoclaving (Figure 4d), demonstrating the stability of this dye and covalent bond under alkaline conditions at elevated temperatures. The crystallinity of the CNF was unaffected by this treatment as observed by solid state NMR (Figure S3). The loss in fluorescence with as received CNF demonstrates that the fluorophore preferentially reacts with phenolic hydroxyl groups of lignin over the aliphatic hydroxyl groups of cellulose glucose units and is highly correlated with the previous studies on lignin extraction from lignocellulosic material [42,43]. Removal of these surface phenols prior to labeling leads to the covalent attachment of the fluorescent probe with the hydroxyl groups of cellulose rather than surface impurities (e.g., lignin or other polyphenols).

**Figure 5:**
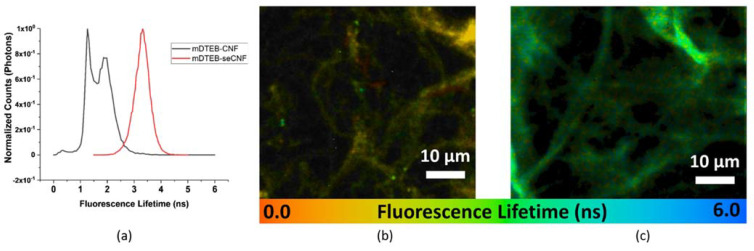
(**a**) The lifetime distributions of mDTEB-CNF (blue line: as received CNF w/mDTEB, red line: SE CNF w/mDTEB) and fluorescence lifetime image of mDTEB labeled CNF (**b**) as received CNF w/mDTEB (**c**) SE CNF w/mDTEB.

Time-correlated single photon counting (TCSPC) fluorescence lifetime imaging microscopy (FLIM) was used to check the quality of commercial CNF and probe the local environment of the mDTEB. The fluorescence lifetime of a fluorophore depends on its molecular environment but, within reasonable limits, not on its concentration [44,45]. A fluorophore that is more mobile will have more opportunities for collisions or other non-radiative energy transfer and will have shorter lifetimes. Free fluorophore will have a shorter lifetime than physisorbed fluorophore, which will have a shorter lifetime than covalently bound fluorophore. In addition, it is expected that a fluorophore bound to lignin, which has random and chaotic linkages and branching leading to more degrees of freedom for a bound fluorophore and large number of aromatic groups capable of quenching fluorescence, will have a shorter lifetime than a fluorophore bound to cellulose. The unique fluorescence lifetime signatures of mDTEB in these various environments was used to distinguish bonded fluorophore–CNF conjugates from fluorophore—lignin, lignin autofluorescence, or nonbonded fluorophore–CNF physisorption. The fluorescence lifetime distributions and ratiometric fluorescence lifetime imaging microscopy (FLIM) images of mDTEB-CNF and mDTEB-seCNF are presented in Figure 5. mDTEB-CNF exhibited a bimodal distribution of lifetimes centered around 1 ns and 2 ns, whereas mDTEB-seCNF exhibited a single, narrow lifetime distribution centered at a longer lifetime of 3.5 ns. In addition, the total photon count for mDTEB-CNF was an order of magnitude lower than that for mDTEB-seCNF.

FLIM analysis also showed that lignin-like impurities, present on the surface of CNF, reduce the fluorescence intensity of mDTEB-CNF via quenching. As a result, the chemical composition and the methods of CNF production affect subsequent studies. Removal of these surface impurities is important for several reasons. First, autofluorescence from these impurities creates interference with or quenching of the dye. Second, even though cellulose constitutes the bulk of the fiber by mass, due to the stronger acidity of phenols over aliphatic alcohols, the dye will preferentially react with the lignin-type impurities on the surface of the cellulose. Lignin is constructed primarily through three hydroxyl-phenylpropane compounds, forming a complex and heterogeneous structure of phenolic moieties [46]. Dye attachment will occur at these phenolic hydroxyls. Third, the impurities are not stable under extremely acidic or alkaline conditions and would likely be removed from the nanofiber during digestion. Additionally, fourth, the fluorescence lifetime of dye bound to these impurities is much shorter and broader than that of cellulose bound dye, leading to difficulties in differentiating bound dye from unreacted, free dye [47]. The lifetime distribution and FLIM image for mDTEB physisorbed to CNF is shown in Figure S4. These impurities can be mostly removed after labeling using the same methods to produce seCNF, however, the fluorescence intensity of the final product is greatly reduced.

**Figure 6:**
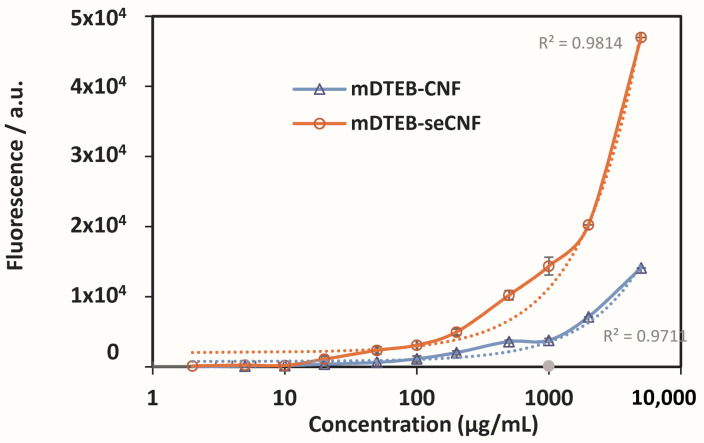
Concentration-dependent fluorescence intensity of the labeled CNF (circle: mDTEB-seCNF, triangle: mDTEB-CNF).

Concentration-dependent fluorescence is important for both in vivo feeding and in vitro kinetics assays, as these assays require several fold dilutions of initial concentrations. In this context, the limit of detection for mDTEB-CNF and mDTEB-seCNF were determined in PBS buffer. mDTEB-seCNF can be detected under physiological conditions at concentration ranging from 2 µg/mL to 2000 µg/mL using a microplate reader (Figure 6). The limit of detection was noted as 3 times the background noise.

The new fluorophore was found to be chemically stable with low detection limits, making it suitable for digestive studies. Before proceeding with the ADME or toxicological studies, it is important to ensure that the labeling process did not alter the surface properties of the cellulose. If the surface were over-labeled, then it would no longer have the same interactions in biological media, it could no longer be considered cellulose, and the toxicology studies would be moot. We examined the surface charges, surface energies, and morphology of the fibers to assess if the labeling significantly altered the cellulose.

**Figure 7:**
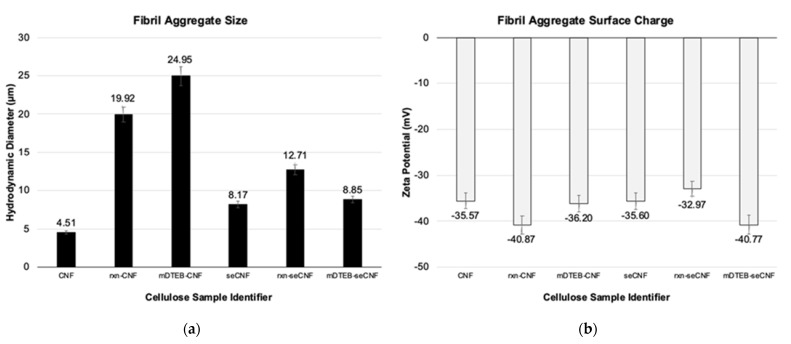
Hydrodynamic particle sizes (**a**) and zeta potential (**b**) of CNF materials measured at 0.1% by mass.

**Figure 8:**
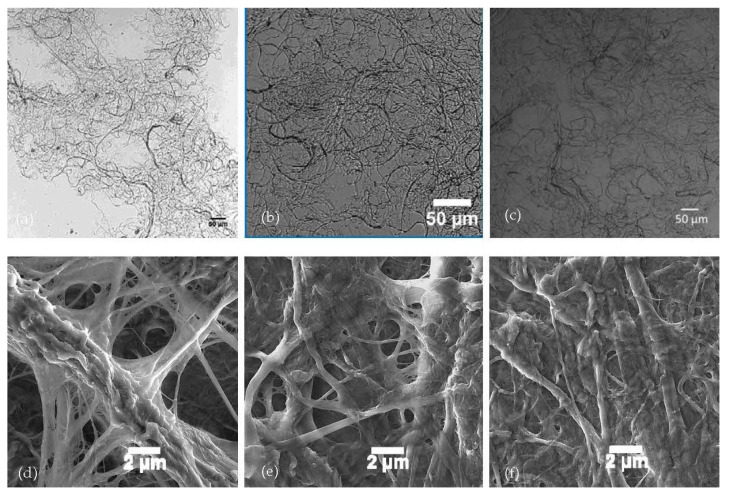
Wide field transmission images of (**a**) as received CNF, (**b**) seCNF, and (**c**) mDTEB-CNF and SEM images of (**d**) as received CNF, (**e**) seCNF, and (**f**) mDTEB-CNF.

Morphological and surface characteristics of the CNF were examined using dynamic light scattering (DLS), zeta potential (ZP), and inverse gas chromatography (iGC). It is well known that alkaline conditions cause the separation and removal of lignin, increases in swelling and porosity, and changes in crystallinity in cellulosic materials [48,49]. Not surprisingly, the basic reaction conditions during the labeling process caused an increase in the apparent particle size of the CNF (Figure 7a). The addition of the dye did not significantly affect the size. The surface extracted CNF did not show the same variation in particle size as was seen with the as received CNF. Although slightly larger than as received CNF, likely due to alkali treatment, additional base treatment through the labeling process did not affect the seCNF size. This suggests that some of the size increase in as received CNF is due to the de-binding of surface-adhered, lignin-like impurities. The surface charges of all samples (Figure 7b) were largely unaffected by the reaction conditions. Wide field optical transmission images (Figure 8a–c) and scanning electron microscopy images (Figure 8d–f) provide additional evidence that the nano morphology is relatively unchanged after surface extraction and labeling.

**Figure 9:**
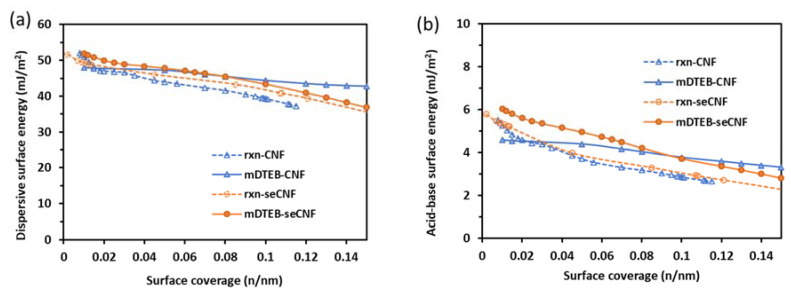
(**a**) Dispersive and (**b**) acid−base surface energy profiles for the freeze-dried samples measured at 30 °C. Fraction surface coverage is the number of moles (n) divided by the moles required to cover a surface monolayer (nm).

The inclusion of iGC in the analysis of the fluorescent CNF affords the opportunity to study the interactions between small probe molecules with different properties (such as octane and acetone) and the surface of solid samples. Linear alkane probes were used to determine the dispersive (van der Waals) interactions, while a series of polar probes was used to determine the acid–base character of the CNM. For these measurements, freeze-dried CNF samples were placed in a chromatographic column as the stationary phase, and solvent probe molecules were injected into the carrier gas stream. The retention time and shape of the chromatogram gives information on surface heterogeneity, surface area, acid–base properties, and surface energy [28]. The column in iGC is filled with the solid sample under investigation (adsorbent) and the mobile phase consists of a probe molecule (adsorbate) with known properties to evaluate the surface of the adsorbent. Most solid samples have a range of surface energies due to defects, accessibility, and trace impurities. By performing experiments over a range of concentrations the heterogeneity of the surface energy can be elucidated. Dispersive and acid–base surface energy profiles for different CNF samples are shown in Figure 9. The surface energy is noticeably higher at low surface coverage for each of the samples, which can be due to surface defects or impurities (Figure 9). As received CNF samples had a dispersive surface energy value (<45 mJ/m^2^) which is similar to the wood-based cellulose nanofibers studied previously. [50] As received CNFs have acid–base and dispersive energy profiles consistent with those found in the literature [51] for CNF produced from wood using mechanical methods. The alkaline treatment used to produce seCNF led to a slight increase in dispersive energy and no appreciable change in acid–base properties. Alkaline treatments are known to change the crystal structure of cellulose [52,53], which may have led to greater accessibility of the non-polar probes to the surface of seCNF. The covalent attachment of mDTEB led to slightly higher dispersive and acid–base energies, whether the CNF was surface extracted or not. The slight changes in surface energy are likely due to the introduction of bulky aromatic-containing moieties. The pyranose ring of the unlabeled CNF, which accounts for the large dispersive energy of cellulose, becomes more accessible with the introduction of mDTEB, leading to a higher number or frequency of non-polar active sites and higher dispersion energy. Aromatic groups add basic interactions, which can account for the slight increase in acid–base properties of the labeled CNF relative to the unlabeled CNF. Although the changes in surface energy are small, these measurements show how small changes in surface groups can greatly affect surface energies and emphasizes the importance of avoiding over-labeling when modifying CNMs.

### 3.2. Toxicity Studies

After demonstrating that the new label was chemically stable, detectable at low concentrations of CNF, and suitable for digestive studies, the toxicity of the labeled CNF was evaluated in an in vitro small epithelial cellular model. In vivo toxicity and biodistribution of labeled CNF were evaluated in a zebrafish model.

**Figure 10:**
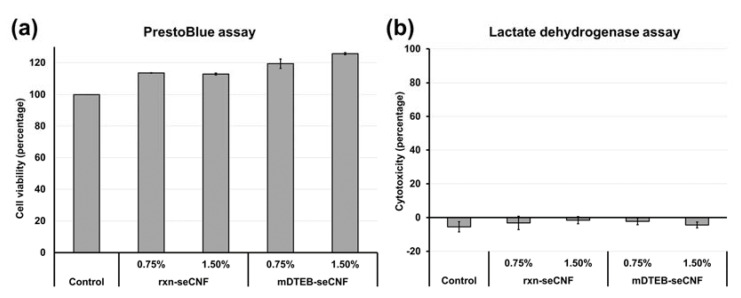
Cytotoxicity of CNF triculture epithelium exposed to digestas of rxn-seCNF or mDTEB-seCNF at starting concentrations of 0.75% and 1.5% by mass. Controls were treated with digestas of water without CNF. (**a**) PrestoBlue assay (n = 4) (**b**) LDH assay (n = 4). Error bars represent ± one standard deviation.

Single-parameter in vitro tests can provide an indication of the relative concentrations at which a substance is toxic, as well as the mechanisms underlying the effects. To simulate the oral exposure of CNFs to the small intestinal epithelium, we applied digestas (from simulated three-phase digestion) of the CNFs (at 0.75% and 1.5% by mass) from the fasting food model (phosphate buffer) to transwell and 96-well versions of a tri-culture small intestinal epithelium model. LDH release (cytotoxicity) and metabolic activity (viability, PrestoBlue) were measured after 24 h. The results of these experiments are summarized in Figure 10a. No significant reduction in cell viability (reduction in the PrestoBlue reagent by mitochondrial reductases) was seen with either unlabeled CNF or mDTEB-seCNF at food concentrations up to 1.5% (by mass). It should be noted that the initial food concentrations are diluted by a factor of 48 during the digestion and preparation of samples (see methods), thus the final concentrations applied to triculture cells were 0.015% and 0.03% by mass. These results indicate that neither labeled nor unlabeled CNF cause significant toxic effects on the small intestinal epithelium after 24 h exposure.

The absence of cytotoxic effects was confirmed by the measurement of LDH release, an indicator of plasma membrane damage, in the triculture epithelial cells exposed to CNF-containing digestas (Figure 10b). LDH release from cells treated for 24 h with CNF-containing digestas was not significantly different than from cells treated with control digestas (without CNF). Together, the PrestoBlue and LDH results suggest that labeled and unlabeled CNF materials are non-toxic in an in vitro small intestinal epithelial model. These findings are in agreement with other in vitro and in vivo studies published on various CNF and CNC materials [8,10,54,55,56].

**Figure 11:**
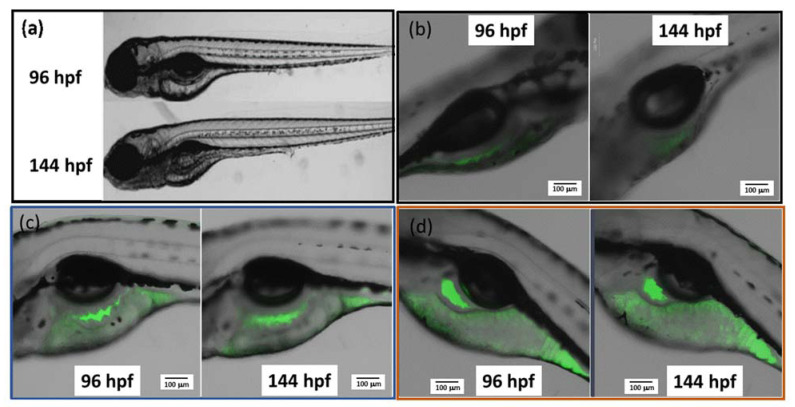
Confocal images of mDTEB-labeled CNF uptake in zebrafish yolks sac after 96 and 144 h post-fertilization (hpf) (**a**) brightfield image of 4-and-6-day-old zebrafish (**b**) control autofluorescence in yolk sac at 96 and 144 hpf (**c**) as received CNF w/mDTEB at 96 and 144 hpf (**d**) SE CNF w/mDTEB at 96 and 144 hpf. Scale bar in panels b–d indicate 100 μm.

In previous sections, we have shown that the fluorophore-labeled CNF exhibits the desired fluorescence, allowing precise imaging by confocal laser scanning microscopy. As such, it may serve as a potential biomarker that allows for the fluorescence-based optical detection of CNF uptake and distribution in living organisms. Zebrafish embryos at 96 h post-fertilization (hpf) were exposed to mDTEB-labeled CNF samples, and fluorescence imaging was performed at 144 hpf which allowed for oral exposure as a result of the mouth gaping behavior [57]. Using this technique, we were able to readily visualize the uptake of the fluorescent CNF within the gastrointestinal (GI) tract and follow its distribution to the anus of the fish (Figure 11). Although there was some naturally occurring autofluorescence within discrete small sections of the yolk sac of control fish (Figure 11b), the fluorescence images revealed the presence of the luminescent mDTEB-CNF and mDTEB-seCNF throughout the yolk sac at a much higher intensity (Figure 11c and d, respectively). There was no embryo mortality as a result of exposure to either of the nanofibers, supporting the low toxicity of mDTEB-CNF towards developing zebrafish following both dermal and oral exposure. In addition, tests with similar concentrations and increasing up to 0.36% by the mass of unlabeled CNF caused no significant toxicity, and no change in toxicity can be noted following conjugation of the materials with the test fluorophores. These results are further supported by previous studies of lignin nanoparticles showing they are of very low toxicity to zebrafish embryos [58]. These results are supporting data that highlight the nontoxicity of the mDTEB-CNF and, as a new kind of nanomaterial based on wood, important for demonstrating their biocompatibility for their future use in food and pharmaceutical applications.

## 4. Conclusions

A two-photon fluorescent probe, meso-DichlorotriazineEthyl Bodipy (mDTEB), based on the BODIPY skeleton, was attached to CNF in aqueous solution for the quantitative tracking CNF in biological media, i.e., the gastrointestinal tract system. The labeled CNF was assessed for its suitability in digestive studies, where environmentally harsh conditions exist. mDTEB-CNF was stable over a wide pH range, from pH 2 to pH 10, and was thermally stable under autoclave conditions. The labeled CNF was detectable to 2 μg/mL. Fluorescence lifetime imaging microscopy showed that lignin-like surface impurities had an impact on the labeling chemistry, indicated by the observation that mDTEB-seCNF had higher fluorescence lifetimes compared to mDTEB-CNF. Removing these impurities prior to labeling was important for maintaining the low detection limit needed for ingestion exposure studies. Surface characterization measurements showed that the label did not significantly alter the hydrodynamic radius of the nanofibers, the surface charges, or the surface energy of the CNF, indicating that the mDTEB-seCNF materials were physicochemically and morphologically representative of commercially produced CNF. The toxicity of CNF was found to be low using the mDTEB-CNF in simulated digestion and in vitro small intestinal epithelial model experiments, and in in vivo zebrafish ingestion and biodistribution analyses.

## Figures and Tables

**Table 1:** Notation used throughout this manuscript.

Abbreviation	Material Description
mDTEB	Synthesized fluorophore, meso-DichloroTriazineEthyl BODIPY
CNF	Cellulose nanofibrils, as received
seCNF	Surface extracted CNF
mDTEB-CNF	mDTEB labeled as received CNF
mDTEB-seCNF	mDTEB labeled surface extracted CNF
rxn-CNF	As received CNF exposed to reaction conditions without presence of mDTEB
rxn-seCNF	Surface extracted CNF exposed to reaction conditions without presence of mDTEB

## Data Availability

The data presented in this study are available on request from the corresponding author. Some data is protected proprietary information.

## Supplementary Materials

The following are available online at https://www.mdpi.com/article/10.3390/nano11041015/s1, Acid soluble lignin (ASL) and acid insoluble lignin (AIL) for as received CNF, CNF treated under labeling alkaline reaction conditions, and surface extracted CNF using an autoclave. Figure S2. Schematic of process used to determine chemical stability of covalent attachment of mDTEB. Figure S3. CPMAS-NMR for CNF and seCNF. Figure S4. The lifetime distribution and fluorescence lifetime image of mDTEB physisorbed onto CNF.

## References

[1] Moon R.J., Schueneman G.T., Simonsen J. (2016). Overview of Cellulose Nanomaterials, Their Capabilities and Applications. Jom.

[2] Bohlmann G.M. (2004). Biodegradable packaging life-cycle assessment. Environ. Prog..

[3] Zhang Y. (2013). Cellulose Nanofibrils. J. Renew. Mater..

[4] Moon R.J., Martini A., Nairn J., Simonsen J., Youngblood J. (2011). Cellulose nanomaterials review: Structure, properties and nanocomposites. Chem. Soc. Rev..

[5] Lavoine N., Desloges I., Dufresne A., Bras J. (2012). Microfibrillated cellulose—Its barrier properties and applications in cellulosic materials: A review. Carbohydr. Polym..

[6] Hubbe M.A., Rojas O.J., Lucia L.A. (2015). Green Modification of Surface Characteristics of Cellulosic Materials at the Molecular or Nano Scale: A Review. BioResources.

[7] Nechyporchuk O., Belgacem M.N., Bras J. (2016). Production of cellulose nanofibrils: A review of recent advances. Ind. Crop. Prod..

[8] DeLoid G.M., Cao X.Q., Molina R.M., Silva D.I., Bhattacharya K., Ng K.W., Loo S.C.J., Brain J.D., Demokritou P. (2019). Toxicological effects of ingested nanocellulose in in vitro intestinal epithelium and in vivo rat models. Environ. Sci.-Nano.

[9] Pradhan S.H., Mulenos M.R., Steele L.R., Gibb M., Ede J.D., Ong K.J., Shatkin J.A., Sayes C.M. (2020). Physical, chemical, and toxicological characterization of fibrillated forms of cellulose using an in vitro gastrointestinal digestion and co-culture model. Toxicol. Res..

[10] Khare S., DeLoid G.M., Molina R.M., Gokulan K., Couvillion S.P., Bloodsworth K.J., Eder E.K., Wong A.R., Hoyt D.W., Bramer L.M. (2020). Effects of ingested nanocellulose on intestinal microbiota and homeostasis in Wistar Han rats. NanoImpact.

[11] Ong K.J., Ede J.D., Pomeroy-Carter C.A., Sayes C.M., Mulenos M.R., Shatkin J.A. (2020). A 90-day dietary study with fibrillated cellulose in Sprague-Dawley rats. Toxicol. Rep..

[12] DeLoid G.M., Sohal I.S., Lorente L.R., Molina R.M., Pyrgiotakis G., Stevanovic A., Zhang R., McClements D.J., Geitner N.K., Bousfield D.W. (2018). Reducing Intestinal Digestion and Absorption of Fat Using a Nature-Derived Biopolymer: Interference of Triglyceride Hydrolysis by Nanocellulose. ACS Nano.

[13] FDA (2007). Redbook 2000: Guidance for Industry and other Stakeholders. Toxicological Principles for the Safety Assessment of Food Ingredients.

[14] Lavin S.R., McWhorter T.J., Karasov W.H. (2007). Mechanistic bases for differences in passive absorption. J. Exp. Biol..

[15] Lopaschuk G.D., Barr R.L., Pierce G.N., Claycomb W.C. (1997). Measurements of fatty acid and carbohydrate metabolism in the isolated working rat heart. Novel Methods in Molecular and Cellular Biochemistry of Muscle.

[16] Robin M.P., O’Reilly R.K. (2015). Strategies for preparing fluorescently labelled polymer nanoparticles. Polym. Int..

[17] Jiang Z., He H., Liu H., Thayumanavan S. (2019). Cellular Uptake Evaluation of Amphiphilic Polymer Assemblies: Importance of Interplay between Pharmacological and Genetic Approaches. Biomacromolecules.

[18] Dong S., Roman M. (2007). Fluorescently Labeled Cellulose Nanocrystals for Bioimaging Applications. J. Am. Chem. Soc..

[19] Abitbol T., Palermo A., Moran-Mirabal J.M., Cranston E.D. (2013). Fluorescent Labeling and Characterization of Cellulose Nanocrystals with Varying Charge Contents. Biomacromolecules.

[20] Navarro J.R.G., Conzatti G., Yu Y., Fall A.B., Mathew R., Edén M., Bergström L. (2015). Multicolor Fluorescent Labeling of Cellulose Nanofibrils by Click Chemistry. Biomacromolecules.

[21] Salari M., Bitounis D., Bhattacharya K., Pyrgiotakis G., Zhang Z., Purington E., Gramlich W., Grondin Y., Rogers R., Bousfield D. (2019). Development & characterization of fluorescently tagged nanocellulose for nanotoxicological studies. Environ. Sci. Nano.

[22] Woodcock J., Fox D.M., Gilman J.W. (2020). Physiologically Stable Fluorophore for Digestion Toxicology Studies. U.S. Patent.

[23] NREL (2012). Determination of Structural Carbohydrates and Lignin in Biomass: Laboratory Analytical Procedure.

[24] TAPPI (2006). Acid-Insoluble Lignin in Wood and Pulp.

[25] TAPPI (2000). Acid-Soluble Lignin in Wood and Pulp.

[26] Beams R., Woodcock J.W., Gilman J.W., Stranick S.J. (2017). Phase Mask-Based Multimodal Superresolution Microscopy. Photonics.

[27] Burnett D.J., Khoo J., Naderi M., Heng J.Y.Y., Wang G.D., Thielmann F. (2012). Effect of Processing Route on the Surface Properties of Amorphous Indomethacin Measured by Inverse Gas Chromatography. AAPS PharmSciTech.

[28] Dorris G.M., Gray D.G. (1980). Adsorption of n-alkanes at zero surface coverage on cellulose paper and wood fibers. J. Colloid Interface Sci..

[29] Donnet J., Park S.-J., Balard H. (1991). Evaluation of Specific Interactions of Solid Surfaces by Inverse Gas Chromatography. Chromatographia.

[30] World Health Organization (2012). The International Pharmacopoeia.

[31] Guo Z., Cao X., DeLoid G.M., Sampathkumar K., Ng K.W., Loo S.C.J., Demokritou P. (2020). Physicochemical and Morphological Transformations of Chitosan Nanoparticles across the Gastrointestinal Tract and Cellular Toxicity in an In Vitro Model of the Small Intestinal Epithelium. J. Agric. Food Chem..

[32] Ström G., Öhgren C., Ankerfors M. (2013). Nanocellulose as an additive in foodstuff. Innventia Rep..

[33] Truong L., Harper S.L., Tanguay R.L. (2011). Evaluation of embryotoxicity using the zebrafish model. Methods Mol. Biol..

[34] Usenko C.Y., Harper S.L., Tanguay R.L. (2008). Fullerene C60 exposure elicits an oxidative stress response in embryonic zebrafish. Toxicol. Appl. Pharmacol..

[35] Ulrich G., Ziessel R., Harriman A. (2008). The Chemistry of Fluorescent Bodipy Dyes: Versatility Unsurpassed. Angew. Chem. Int. Ed..

[36] Sola-Llano R., Bañuelos J., Bañuelos-Prieto J., Llano R.S. (2018). Introductory Chapter: BODIPY Dye, an All-in-One Molecular Scaffold for (Bio)Photonics. BODIPY Dyes—A Privilege Molecular Scaffold with Tunable Properties.

[37] Gonçalves M.S.T. (2009). Fluorescent Labeling of Biomolecules with Organic Probes. Chem. Rev..

[38] Marfin Y.S., Solomonov A.V., Timin A.S., Rumyantsev E.V. (2017). Recent Advances of Individual BODIPY and BODIPY-Based Functional Materials in Medical Diagnostics and Treatment. Curr. Med. Chem..

[39] Kolemen S., Akkaya E.U. (2018). Reaction-based BODIPY probes for selective bio-imaging. Coord. Chem. Rev..

[40] Vodyanova O.S., Kochergin B.A., Usoltsev S.D., Marfin Y.S., Rumyantsev E.V., Aleksakhina E.L., Tomilova I.K. (2018). BODIPY dyes in bio environment: Spectral characteristics and possibilities for practical application. J. Photochem. Photobiol. A Chem..

[41] Kaur P., Singh K. (2019). Recent advances in the application of BODIPY in bioimaging and chemosensing. J. Mater. Chem. C.

[42] Sasmal S., Mohanty K., Kumar S., Sani R.K. (2018). Pretreatment of Lignocellulosic Biomass Toward Biofuel Production. Biorefining of Biomass to Biofuels: Opportunities and Perception.

[43] Kim S., Holtzapple M.T. (2006). Delignification kinetics of corn stover in lime pretreatment. Bioresour. Technol..

[44] Berezin M.Y., Achilefu S. (2010). Fluorescence Lifetime Measurements and Biological Imaging. Chem. Rev..

[45] Roberts M.S., Dancik Y., Prow T.W., Thorling C.A., Lin L.L., Grice J.E., Robertson T.A., König K., Becker W. (2011). Non-invasive imaging of skin physiology and percutaneous penetration using fluorescence spectral and lifetime imaging with multiphoton and confocal microscopy. Eur. J. Pharm. Biopharm..

[46] Du L., Wang Z., Li S., Song W., Lin W. (2013). A Comparison of Monomeric Phenols Produced from Lignin by Fast Pyrolysis and Hydrothermal Conversions. Int. J. Chem. React. Eng..

[47] Woodcock J.W., Stranick S.J., Patel I., Fox D.M., Gilman J.W. A new fluorescent label for use in carbohydrate nanomaterial in vivo studies.

[48] Al-Shamary E., Khalaf A. (2013). Influence of Fermentation Condition and Alkali Treatment on the Porosity and Thickness of Bacterial Cellulose Membranes. TOJSAT.

[49] Budtova T., Navard P. (2016). Cellulose in NaOH–water based solvents: A review. Cellulose.

[50] Siddiqui N., Mills R.H., Gardner D.J., Bousfield D. (2011). Production and Characterization of Cellulose Nanofibers from Wood Pulp. J. Adhes. Sci. Technol..

[51] Sacui I.A., Nieuwendaal R.C., Burnett D.J., Stranick S.J., Jorfi M., Weder C., Foster E.J., Olsson R.T., Gilman J.W. (2014). Comparison of the Properties of Cellulose Nanocrystals and Cellulose Nanofibrils Isolated from Bacteria, Tunicate, and Wood Processed Using Acid, Enzymatic, Mechanical, and Oxidative Methods. ACS Appl. Mater. Interfaces.

[52] Nishiyama Y., Kuga S., Okano T. (2000). Mechanism of mercerization revealed by X-ray diffraction. J. Wood Sci..

[53] Duchemin B.J.C. (2015). Mercerisation of cellulose in aqueous NaOH at low concentrations. Green Chem..

[54] Harper B.J., Clendaniel A., Sinche F., Way D., Hughes M., Schardt J., Simonsen J., Stefaniak A.B., Harper S.L. (2016). Impacts of chemical modification on the toxicity of diverse nanocellulose materials to developing zebrafish. Cellulose.

[55] Bitounis D., Pyrgiotakis G., Bousfield D., Demokritou P. (2019). Dispersion preparation, characterization, and dosimetric analysis of cellulose nano-fibrils and nano-crystals: Implications for cellular toxicological studies. NanoImpact.

[56] Lopes V.R., Strømme M., Ferraz N. (2020). In Vitro Biological Impact of Nanocellulose Fibers on Human Gut Bacteria and Gastrointestinal Cells. Nanomaterials.

[57] van Pomeren M., Brun N.R., Peijnenburg W.J.G.M., Vijver M.G. (2017). Exploring uptake and biodistribution of polystyrene (nano)particles in zebrafish embryos at different developmental stages. Aquat. Toxicol..

[58] Nix C.E., Harper B.J., Conner C.G., Richter A.P., Velev O.D., Harper S.L. (2018). Toxicological Assessment of a Lignin Core Nanoparticle Doped with Silver as an Alternative to Conventional Silver Core Nanoparticles. Antibiotics.

