# Supplementary material for: Fluorescently Labeled Cellulose Nanofibers for Environmental Health and Safety Studies
Source: Nanomaterials (Basel). 2021 Apr 15;11(4):1015. doi: 10.3390/nano11041015 (PMC8071547; doi:10.3390/nano11041015)
Supplement: Supplementary file 1 [file nanomaterials-11-01015-s001.zip › nanomaterials-1140091-SI.pdf]

Supplementary Materials

Fluorescently labeled cellulose nanofibers for environmental health and safety studies

Ilabahen Patel[1], Jeremiah Woodcock[2], Ryan Beams[2], Stephen J. Stranick[2], Ryan Nieuwendaal[2], Jeffrey W. Gilman[2], Marina R. Mulenos[3], Christie M. Sayes[3], Maryam Salari[4], Glen DeLoid[4], Philip Demokritou[4], Bryan Harper[5], Stacey Harper[5], Kimberly J. Ong[6], Jo Anne Shatkin[6], and Douglas M. Fox[1,*]

The content of acid-insoluble (Klason) and acid-soluble lignin (ASL) was determined from CNFs samples according to TAPPI T222 om-06 and TAPPI UM250, respectively. CNFs was hydrolyzed using sulfuric acid (72% by mass) for 1 h at 30°C, after hydrolysis samples diluted (3% by mass) using deionized water and subsequently autoclaved at 121°C for 1 h. The resulting solution was cooled to room temperature and the precipitate was filtered, dried, and weighed to get the acid-insoluble residues (AIR) named as Klason lignin content as 1.60% for as received CNFs, 1.43% for control CNFs (sample treated under alkaline reaction condition without fluorescent probe), and 1.13% for surface extracted CNFs (Figure S1). The acid-soluble lignin (ASL) was calculated as 0.82%, and 0.84% for as received and surface extracted CNFs, respectively, by measuring absorbance at 205 nm with a spectrophotometer. The loss in lignin content for the two samples treated under different alkaline conditions is attributed to surface bound lignin and/or lignin fragment contaminants.

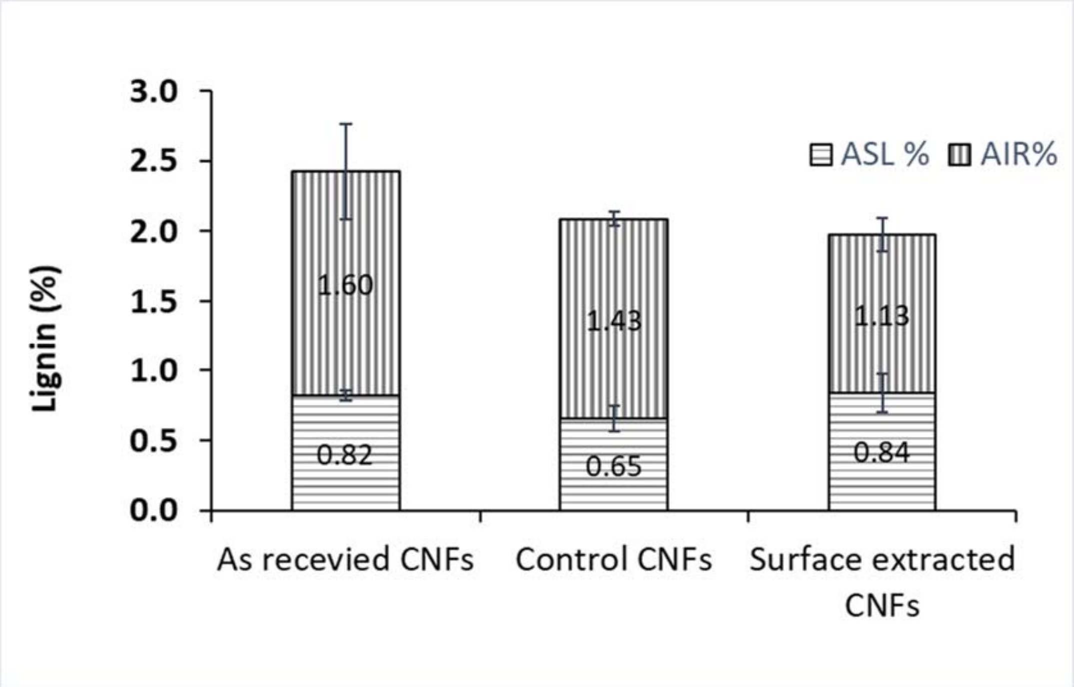

Figure S1. Acid soluble lignin (ASL) and acid insoluble lignin (AIL) for as received CNF, CNF treated under labeling alkaline reaction conditions, and surface extracted CNF using an autoclave.

To test the stability of the mDTEB – CNF bond, samples were autoclaved in base. A sample of the fibers were removed, washed with de-ionized water, and spin coated onto glass cover slips. The supernatant was filtered through a 0.2 mm syringe filter and added to a cuvette. A schematic is shown in Figure S2.

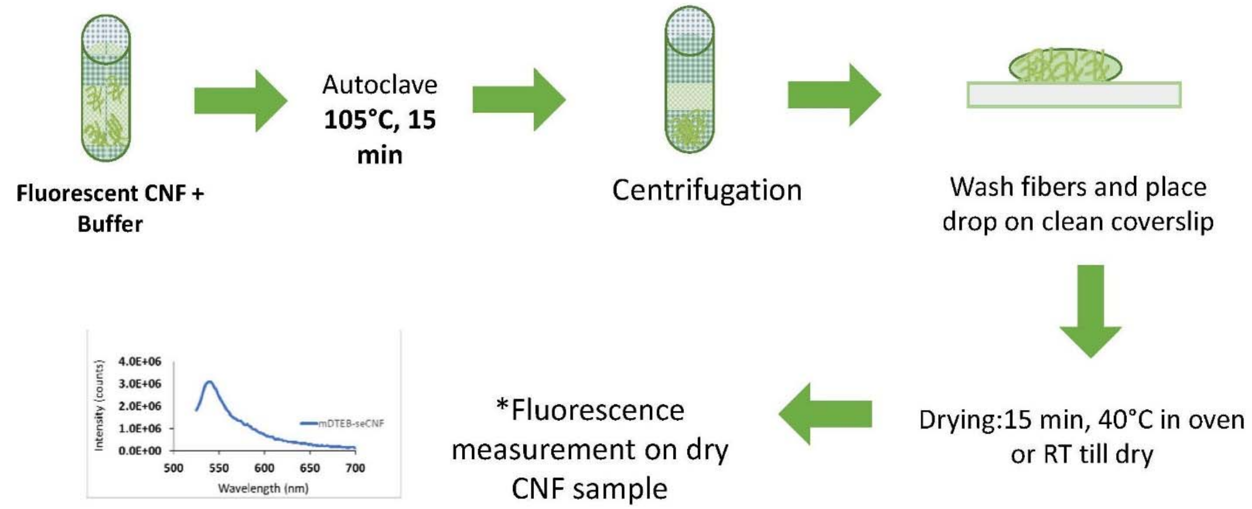

Figure S2. Schematic of process used to determine chemical stability of covalent attachment of mDTEB.

The crystallinity of the CNF and seCNF were compared using 13C Cross-Polarization Magic Angle Spinning (CPMAS) Nuclear Magnetic Resonance (NMR). Solid-state NMR experiments were performed at 100 MHz (2.35 T) on a Tecmag Apollo spectrometer, ultrawide bore Nalorac magnet, and in-house designed 7.5 mm double resonance magic angle spinning probe. Each sample (≈100 mg freeze-dried nanocellulose) was pressed into a 6 mm × 7 mm disk, placed into a Macor rotor, and spun at 3800 ± 100 Hz. CPMAS NMR experiments were performed with the following conditions: 25.19 MHz 13C frequency, 100.16 MHz 1H frequency, 3.2 μs 1H π/2 pulse, 2 ms contact time, 72 kHz 13C contact pulse, 68 kHz 1H contact pulse, 78 kHz continuous wave (cw) decoupling, 100 μs dwell time, 600 data points with 15784 zero filling points, 2048 to 8196 scans, 3 s recycle delay, and 8192 to 32768 transients. The 1H cw decoupling frequency was set to 1.3 ppm relative to tetramethylsilane (TMS) at 0 ppm. The frequency scale of the 13C NMR spectra were referenced to TMS (0 ppm) utilizing adamantane as a secondary reference. Figure S3 compares the spectra for CNF and seCNF. Linear combinations of the 13C CPMAS w/ and w/out T1rH filter allows for deconvolution of ordered and disordered cellulose spectra. The ordered and disordered spectra are self-consistent across the series of 4 samples. No appreciable change in cellulose order after delignification step.

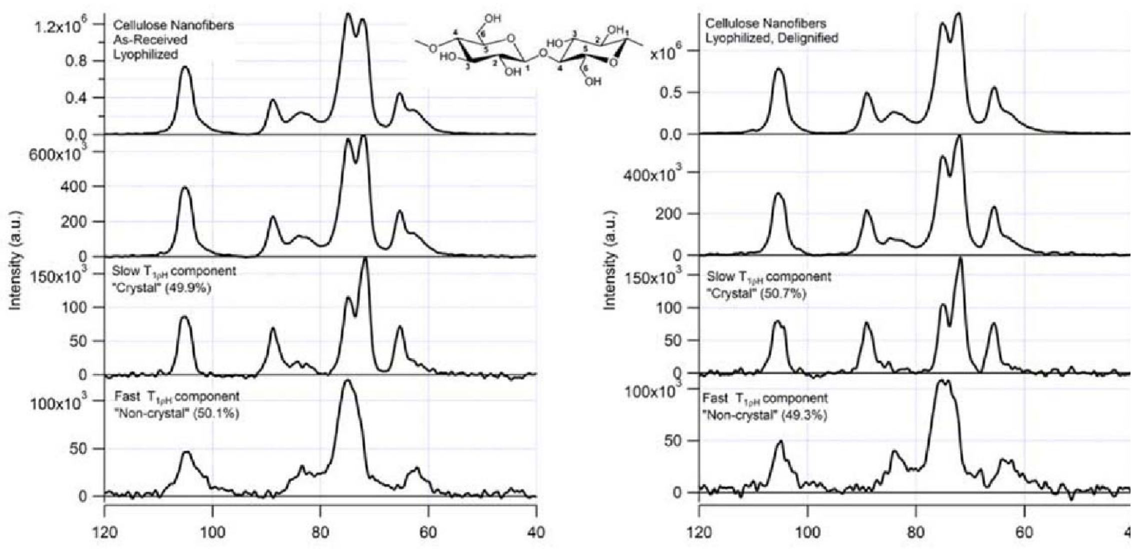

Figure S3. CPMAS-NMR for CNF and seCNF.

The fluorescence lifetime distribution and false color fluorescence lifetime image for mDTEB absorbed onto CNF is shown in Figure S4. mDTEB was placed in alkaline water for several hours to ensure all chloride moieties had been fully hydrolyzed to non-reactive hydroxides. The broad distribution at very short lifetimes is typical for free dye and physisorbed dye materials.

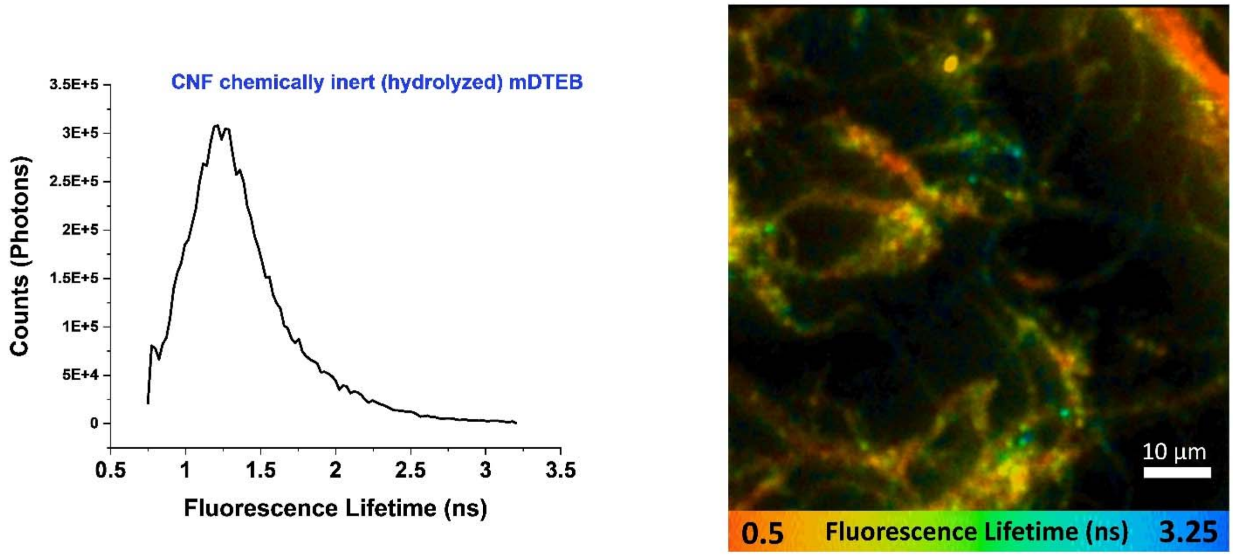

Figure S4. The lifetime distribution and fluorescence lifetime image of mDTEB physisorbed onto CNF.
